# A New Allele of the *SPIKE1* Locus Reveals Distinct Regulation of Trichome and Pavement Cell Development and Plant Growth

**DOI:** 10.3389/fpls.2019.00016

**Published:** 2019-01-24

**Authors:** Shuang Liang, Xuying Yang, Meng Deng, Jun Zhao, Jingxia Shao, Yafei Qi, Xiayan Liu, Fei Yu, Lijun An

**Affiliations:** State Key Laboratory of Crop Stress Biology for Arid Area, College of Life Sciences, Northwest A&F University, Yangling, China

**Keywords:** branching, cell morphogenesis, cytoskeleton, nucleus positioning, *SPIKE1*, trichome

## Abstract

The single-celled trichomes of *Arabidopsis thaliana* have long served as an elegant model for elucidating the mechanisms of cell differentiation and morphogenesis due to their unique growth patterns. To identify new components in the genetic network that governs trichome development, we carried out exhaustive screens for additional Arabidopsis mutants with altered trichome morphology. Here, we report one mutant, *aberrantly branched trichome1-1* (*abt1-1*), with a reduced trichome branching phenotype. After positional cloning, a point mutation in the *SPIKE1* (*SPK1*) gene was identified in *abt1-1*. Further genetic complementation experiments confirmed that *abt1-1* is a new allele of *SPK1*, so *abt1-1* was renamed as *spk1-7* according to the literatures. *spk1-7* and two other *spk1* mutant alleles, covering a spectrum of phenotypic severity, highlighted the distinct responses of developmental programs to different *SPK1* mutations. Although null *spk1* mutants are lethal and show defects in plant stature, trichome and epidermal pavement cell development, only trichome branching is affected in *spk1-7*. Surprisingly, we found that *SPK1* is involved in the positioning of nuclei in the trichome cells. Lastly, through double mutant analysis, we found the coordinated regulation of trichome branching between *SPK1* and two other trichome branching regulators, *ANGUSTIFOLIA* (*AN*) and *ZWICHEL* (*ZWI*). *SPK1* might serve for the precise positioning of trichome nuclei, while *AN* and *ZWI* contribute to the formation of branch points through governing the cMTs dynamics. In summary, this study presented a fully viable new mutant allele of *SPK1* and shed new light on the regulation of trichome branching and other developmental processes by *SPK1*.

## Introduction

The differentiation of distinct cell types is one of the most fundamental features of multicellular organisms (Lee and Schiefelbein, 2002). In higher plants, plant trichomes have served as an amazingly effective paradigm for the investigation of cell differentiation due to their conspicuous presence and defined developmental patterns (Hülskamp et al., 1994; Tominaga-Wada et al., 2011; Grebe, 2012; Ivakov and Persson, 2013; Pattanaik et al., 2014). In model plant *Arabidopsis thaliana*, leaf trichomes are unicellular structures usually bearing three or four branches (Hülskamp et al., 1994), and numerous pathways that control trichome branching have been identified in the last two decades (Folkers et al., 1997; Hülskamp, 2004; Guimil and Dunand, 2007; Ishida et al., 2008; Yang and Ye, 2013).

Generally, the number of trichome branches is closely linked with plant cell cycle control (Schnittger and Hülskamp, 2002; Kasili et al., 2010; Yang and Ye, 2013). During Arabidopsis trichome development, once the cell fate is determined, trichome progenitor cells switch from mitotic division to endoreplication and typically undergo 4 rounds of endoreplication, giving the trichome cells an average of 32C (C equals haploid DNA content per nucleus) in the nuclear DNA content (Folkers et al., 1997; Hülskamp, 2004). A myriads of mutants including *kaktus, spindly, polycomb, triptychon, rastifari*, and *rpt2a* showed extended endoreplication and elevated ploidy, and thus displayed hyperbranched trichomes, suggesting a certain degree of correlation between trichome branching and endoreplication (Szymanski and Marks, 1998; Perazza et al., 1999; Sonoda et al., 2009). However, the correlation between branching and ploidy and nuclear DNA content cannot be extrapolated to all trichome branching mutants, implicating that additional pathways are involved in the elaboration of trichome branching (Ilgenfritz et al., 2003).

Pharmacological and molecular evidences suggest that the organization and dynamics of cortical microtubules (cMTs) are also intimately related with trichome branching determination (Oppenheimer et al., 1997; Mathur and Chua, 2000; Kirik et al., 2002a,b; Abe et al., 2004). During trichome morphogenesis, the arrangement of cMTs changes dramatically at branching points (Mathur and Chua, 2000). Mutations in genes involved in the formation of α/β-tubulin heterodimers or cMTs dynamics often lead to altered trichome branching (Oppenheimer et al., 1997; Burk et al., 2001; Kirik et al., 2002a,b; Abe et al., 2004; Abe and Hashimoto, 2005). Dominant-negative mutations in *α-tubulin 4* (*TUA4*) and *TUA6* genes display abnormal arrangement and destabilization of cMTs and show a reduction in trichome branching (Thitamadee et al., 2002; Abe et al., 2004). In contrast, certain mutant forms of *TUA6* render cMTs more polymerization-prone and promote more branched trichomes formed (Abe and Hashimoto, 2005). Additional factors that are functionally associated with cMTs also play roles in trichome branching determination. *ANGUSTIFOLIA* (*AN*) encodes a C-terminal binding proteins/brefeldin ADP-ribosylated substrates (CtBP/BARS) related protein (Folkers et al., 2002; Kim et al., 2002), and the Arabidopsis *an* mutant showed an abnormal organization of cMTs and predominantly two-branched trichomes (Folkers et al., 2002). *ZWICHEL* (*ZWI*) encodes a member of the unique Kinesin-like Calmodulin-Binding Protein (KCBP) family that can directly bind to cMTs (Reddy et al., 1996; Oppenheimer et al., 1997). Loss of function of *ZWI* leads to the plants with two-branched and short stalk trichomes. Interestingly, *ZWI* could interact genetically and physically with *AN*, suggesting a role for *AN* in cMTs mediated intracellular trafficking (Reddy et al., 1996; Oppenheimer et al., 1997; Folkers et al., 2002). Recent results showed that *ZWI* may serve as a hub to integrate and coordinate cMTs and actin cytoskeleton to achieve the cytoskeletal configuration necessary for trichome development (Tian et al., 2015).

Despite the tremendous progress in our understanding of the process, the full regulatory network of plant trichome development has not yet to be established. To identify additional genes and processes that regulate trichome development, we carried out large-scale forward genetic screening in *A. thaliana* and identified a mutant which we named *abt1-1*. Through map-based cloning, we cloned the *ABT1* locus and identified a G to A mutation in the *SPK1* gene. Genetic and phenotypic analyses further confirmed that *ABT1* is *SPK1 gene*, which encodes a member of the CDM (named after the *Caenorhabditis elegans* CED-5, human DOCK180, and *Drosophila melanogaster* myoblast city) family proteins that functions as a guanine nucleotide exchange factor (GEF) (Kiyokawa et al., 1998; Bompard and Caron, 2004). Taking advantage of the three *SPK1* mutant alleles with varied severity, we demonstrated that the developmental processes regulated by *SPK1* show differential responses to these mutations, revealing a previous unknown mode of plant responses to *SPK1*. More importantly, we determined that *SPK1* is involved in the regulation of nucleus positioning in the trichome cell. Genetic interaction analyses established that *SPK1* interact genetically with *AN* and *ZWI* in the regulation of trichome branch formation. In summary, our findings provide new insight in the functions of *SPK1* and the modes of regulation of plant growth and development by *SPK1*.

## Materials and Methods

### Plant Materials and Growth Conditions

*Arabidopsis thaliana* materials used in this study are all in the *Columbia-0* (Col) background unless indicated otherwise. Ethyl methanesulfonate (EMS)-mutagenesis was carried out in the *abs3-1D* mutant background following previously described procedures (Kim et al., 2006). *abs3-1D* has been described by Wang et al. (2015). The *spk1-7* line described in this study was isolated in the M2 mutant pool. The *abs3-1D* mutant background does not influence the trichome branching defects of *spk1-7* and was removed by backcrossing the original mutant with Col wild type. Two additional rounds of backcross were performed prior to further analysis of *spk1-7*. T-DNA insertional lines for *SPK1* (*SALK_136776C, spk1-5*; *SAIL_520H04, spk1-6*), *AN* (*SALK_026489, an*), *ZWI* (*SALK_017886, zwi*), and a marker line for microtubule arrays *GFP-TUB6* (*CS6550*) were obtained from the Arabidopsis Biological Resource Center (ABRC). The T-DNA insertion sites and homozygous mutants for these lines were confirmed by PCR and sequencing. All primers used in this study are listed in Supplementary Table S1. Arabidopsis seeds were sowed on commercial soil mix (Pindstrup, Denmark) and grown at 22°C under continuous illumination (∼100 μmol m^-2^ s^-1^).

### Phenotypic Characterization of *SPK1* Alleles

The trichome branching phenotypes were analyzed as described in An et al. (2011). In brief, the third and fifth rosette leaves of two-week-old Arabidopsis plants were examined with a SZ61sterescope (Olympus). Numbers of branches of all the trichomes on each leaf were counted and recorded. For each genotype, at least 16 plants were used in the statistical analysis. Student’s *t*-test was used to assess the difference between wild type and mutants. The experiments were repeated as least twice.

For scanning electron microscopy (SEM), fresh leaves were taped on the sample stages, and directly viewed and photographed using a tabletop SEM TM3030 (Hitachi, Japan).

To visualize the outlines of pavement cells, cotyledons of one-week-old seedlings were stained in propidium iodide (PI) (1 mg⋅ml^-1^ in H_2_O) for 5 min and washed multiple times in deionized water. Stained cotyledons were mounted in water and examined with confocal microscopy (Nikon A1) using the following setting: Ex, 561 nm; Em: 595/50 nm.

### Map-Based Cloning

Map-based cloning was conducted according to Lukowitz et al. (2000). *spk1-7* was crossed with Landsberg *erecta* (L*er*) to generate an F2 mapping population. Bulked segregant analysis first mapped *spk1-7* mutation to a region near the Indel markers FCA2#1 and F17L22#1 on chromosome IV. Additional molecular markers were used to fine map the *spk1-7* mutation. Detailed information of molecular markers used in this study has been described in Liu et al. (2010). Candidate genes in the final interval were sequenced to determine the mutation sites.

### RNA Extraction and Real-Time Quantitative RT-PCR

Total RNA was isolated from pooled samples of one-week-old seedlings using the TRIzol reagent (Invitrogen) following the manufacturer’s instructions. First strand cDNAs were synthesized from 1 μg of DNase I treated total RNA using the Transcriptor First Strand cDNA Synthesis Kit (Roche). Real-time PCRs were performed with the SYBR green PCR mix (Roche), and running on a Bio-Rad CFX96 Real-Time PCR system. Relative expression levels of the target genes were calculated with 2^-ΔCt^. The *ACTIN2* gene was used as the internal control. Primers for real-time quantitative RT-PCR analysis were listed in Supplementary Table S1.

### Trichome Nuclei Position Analysis

The third and fifth rosette leaves from three-week old wild type and mutant plants were used in trichome cell nuclei position analysis. Leaves were fixed and cell nuclei were stained by fluorescent dye 4′,6-Diamidino-2-phenylindole dihydrochloride (DAPI), then the nuclei of the DAPI stained trichome cells were observed under the a DM5000B fluorescence microscope (Leica), and the nuclei positions of the cells were captured and recorded. Statistical analyses were performed based on the observation and at least 30 independent trichome cells of each genotype were used.

### Construction of Double Mutants

To generate the *spk1-7 an* and *spk1-7 zwi* double mutants, *spk1-7* was crossed with *an* and *zwi*, respectively. Genotypes of the F1 and F2 progeny were determined by PCR using gene specific primers. Trichome phenotypes were assesses at F2 generation after genotyping. The same strategies were used to construct the *spk1-5 an, spk1-5 zwi, spk1-6 an, spk1-6 zwi* double mutants, and *spk1-7 an zwi* triple mutants, respectively.

### Microscopy and Image Processing

For the fluorescence observation of the cMTs, a marker line *CS6550* which carries a *GFP-TUB6* construct was crossed to the different mutant background and the double mutants were selected by genomic PCR and GFP fluorescence detection. Seeds were sowed into the soil and the cotyledons of 4-day-old seedlings were observed under a spinning disk confocal microscope equipped with lasers for GFP (Nikon A1). Images were stored and processed with the Adobe Photoshop 7.0 program. For the quantitative analysis of the cMTs organization, the microtubule angles were measured by Image J software according to Yao et al. (2008). The value of microtubules angles of that parallel to the cell’s longitudinal axis were defined as 0°, while the value of those were perpendicular to the cell’s longitudinal axis were defined as 90°. The cMTs anisotropy was evaluated according to Boudaoud et al. (2014), and the following convention was used: the anisotropy score 0 for no order (purely isotropic arrays) and 1 for perfectly ordered (purely anisotropic arrays).

## Results

### The Isolation of the *abt1-1* Mutant

**FIGURE 1:**
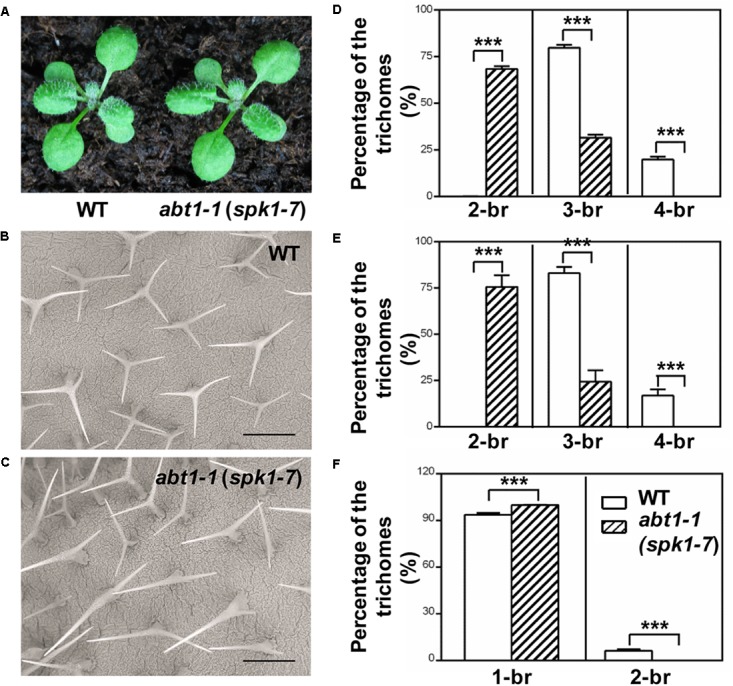
Phenotypes of WT and *abt1-1* (*spk1-7*) plants. **(A)** The overall growth phenotypes of two-week-old wild type (WT) and *abt1-1* (*spk1-7*). **(B,C)** Examination of the trichome branching phenotypes on the fifth rosette leaves of three-week-old WT **(B)** and *abt1-1* (*spk1-7*) **(C)** by SEM. Bars are 250 μM. **(D–F)** Quantification of the trichome branching phenotypes of WT and *abt1-1* (*spk1-7*) on the third rosette leaves **(D)**, the fifth rosette leaves **(E)** and the main inflorescence stems **(F)**. Data were presented as mean ± SD of three biological replicates. ^∗∗∗^*p* < 0.001. 2/3/4-br for two/three/four-branched trichomes, respectively.

In our ongoing effort to identify new factors that define trichome morphology, we carried out large-scale EMS-mutagenesis and a trichome branching defective mutant, designated *abt1-1* was isolated. The overall growth and development of *abt1-1* was not significantly altered compared with the wild type, but displayed a conspicuous trichome branching defect (Figures 1A–C). The majority of leaf trichomes had three branches in wild type while two-branched trichomes became the predominant form in *abt1-1* leaves (Figures 1B–E). Notably, the reduced trichome branching phenotype in *abt1-1* was more pronounced in the fifth rosette leaves than in the third rosette leaves as we observed an average of 68% of two-branched trichomes in the third leaves (Figure 1D) versus an average of 76% of two-branched in the fifth leaves (Figure 1E). This observation suggests that the trichome hypobranching defect in *abt1-1* was developmentally associated. Consistent with the leaf trichome branching phenotype, trichomes on the main inflorescence stem also had fewer branches in *abt1-1* compared to those in the wild type (Figure 1F). Genetic analysis indicated that *abt1-1* behaved as a single recessive mutation. These data suggest that *ABT1* functions as a positive regulator of trichome branching.

### *abt1-1* Is a New Mutant Allele of *SPK1*

**FIGURE 2:**
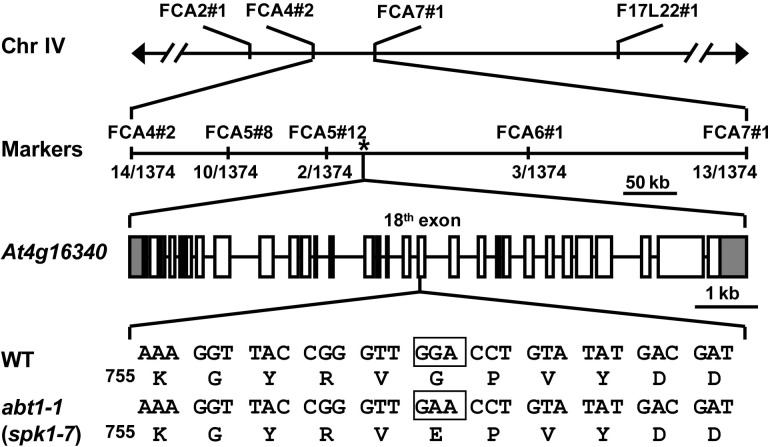
Map-based cloning of *abt1-1* (*spk1-7*). Schematic representation of the map-based cloning of *abt1-1* (*spk1-7*) as described in Methods. *abt1-1* (*spk1-7*) mutation was linked to markers FCA2#1 and F17L22#1 on chromosome IV. Numbers of recombinants were shown under each marker. The asterisk indicated the position of *SPK1* gene, *At4g16340*. In the gene model, boxes and solid lines represent the exons and introns, respectively. The 5′ and 3′ untranslated regions were shown as shaded boxes. The boxed nucleotides below the gene model indicated the exact position of the mutation site.

To further investigate the function of *ABT1* on regulating trichome branching, we carried out map-based cloning. Initial bulked segregant analyses placed the *ABT1* locus near the molecular markers FCA2#1 and F17L22#1 on chromosome IV (Figure 2). Further fine mapping narrowed down the physical interval harboring the *abt1-1* mutation to a ∼200 kb region between molecular markers FCA5#12 and FCA6#1 (Figure 2). We examined all the annotated genes in this interval to identify known loci in which mutations could lead to reduced trichome branching phenotype. Interestingly, T-DNA insertional mutants of one locus, *SPK1*/*At4g16340*, was reported to have predominantly one-branched trichomes (Qiu et al., 2002; Basu et al., 2008). To test whether the mutation in *SPK1* caused the trichome branching defects of *abt1-1*, genomic region of the *SPK1* locus was amplified and sequenced, and a single G to A transition mutation was identified at the +5,893 position (from the ATG start codon) in the *SPK1* locus in *abt1-1* mutant background (Figure 2). Theoretically, this mutation would convert a deduced glycine codon (GGA, Gly760) to a codon for glutamate (GAA, Glu760) (Figure 2).

**FIGURE 3:**
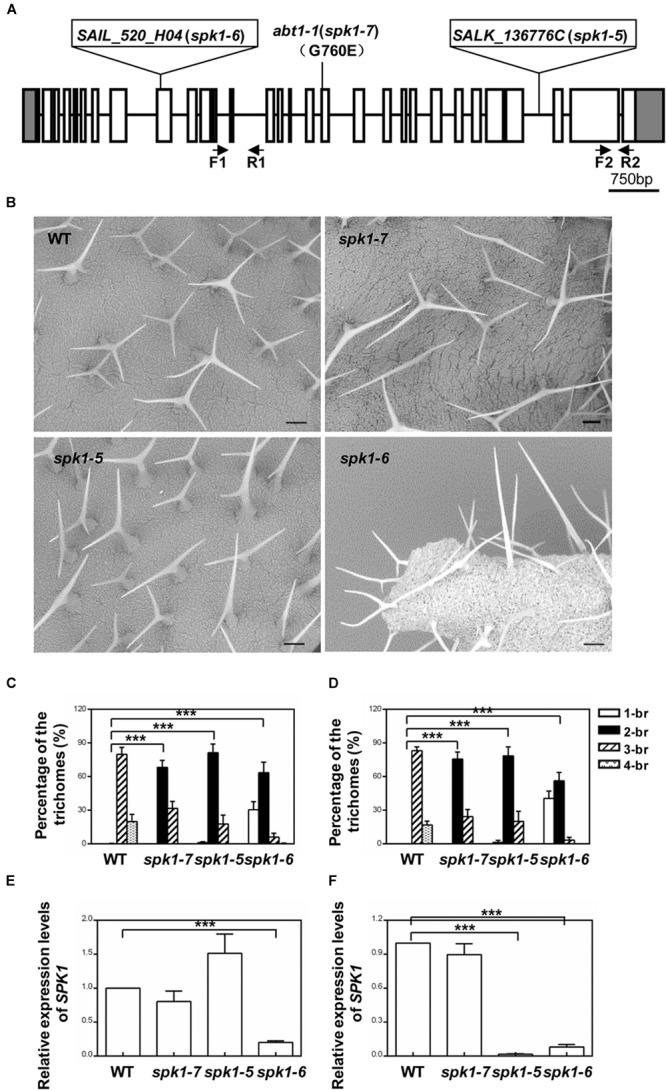
Analysis of the trichome branching phenotypes of various *SPK1* alleles. **(A)** Schematic representation of the mutation sites in *abt1-1* (*spk1-7*), *spk1-5* (*SALK_136776C*), and *spk1-6* (*SAIL_520H04*). **(B)** Trichome branching phenotypes on the fifth rosette leaves of three-week-old WT, *spk1-7, spk1-5*, and *spk1-6* examined by SEM. Bars are 250 μM. **(C,D)** Quantification of the trichome branching phenotypes of WT, *spk1-7, spk1-5*, and *spk1-6* on the third rosette leaf **(C)** and the fifth rosette leaf **(D)**. **(E,F)** Real-time quantitative RT-PCR analysis of transcript accumulations of *SPK1* in different *SPK1* alleles with primers F1 coupled with R1 spanning the 11th–13th exon **(E)**, and primers F2 coupled with R2 covering the 29th exon **(F)**. Data were presented as mean ± SD of three biological replicates. ^∗∗∗^*p* < 0.001. 1/2/3/4-br represented one/two/three/four-branched trichomes, respectively.

*SPK1* genomic region spans13,133 bp, consisting of 30 exons and encodes a predicted protein of 1,830 amino acids. To verify that *abt1-1* indeed represents a new mutant allele of *SPK1*, two *SPK1* alleles, *SALK_136776C* and *SAIL_520H04*, were obtained from ABRC. Through PCR and genomic sequencing, we confirmed that the T-DNAs were inserted in the 27th intron and the in the ninth exon of the *SPK1* gene in *SALK_136776C* and *SAIL_520H04*, respectively, (Figure 3A). SEM examination and quantification of trichome branching on the third and fifth rosette leaves of homozygous *SALK_136776C* and *SAIL_520H04* lines clearly showed that they also display trichome hypobranching phenotypes similar to those of *abt1-1* (Figures 3B–D). *SALK_136776C* showed a slightly stronger branching defect than *abt1-1*. The percentage of two-branched trichomes is higher in *SALK_136776C* compared to that of *abt1-1* (Figures 3C,D). Occasionally, unbranched trichomes could be also observed in *SALK_136776C* (Figures 3C,D). *SAIL_520H04* showed the strongest trichome branching defect, with an average of ∼30% unbranched trichomes (Figures 3C,D). These observations were consistent with the trichome branching defects in other *spk1* alleles reported in previous studies (Qiu et al., 2002; Basu et al., 2008). Moreover, the extent of trichome branching reduction observed in *abt1-1, SALK_136776C* and *SAIL_520H04* agrees in general with the natures of the three mutations (Figures 3E,F).

**FIGURE 4:**
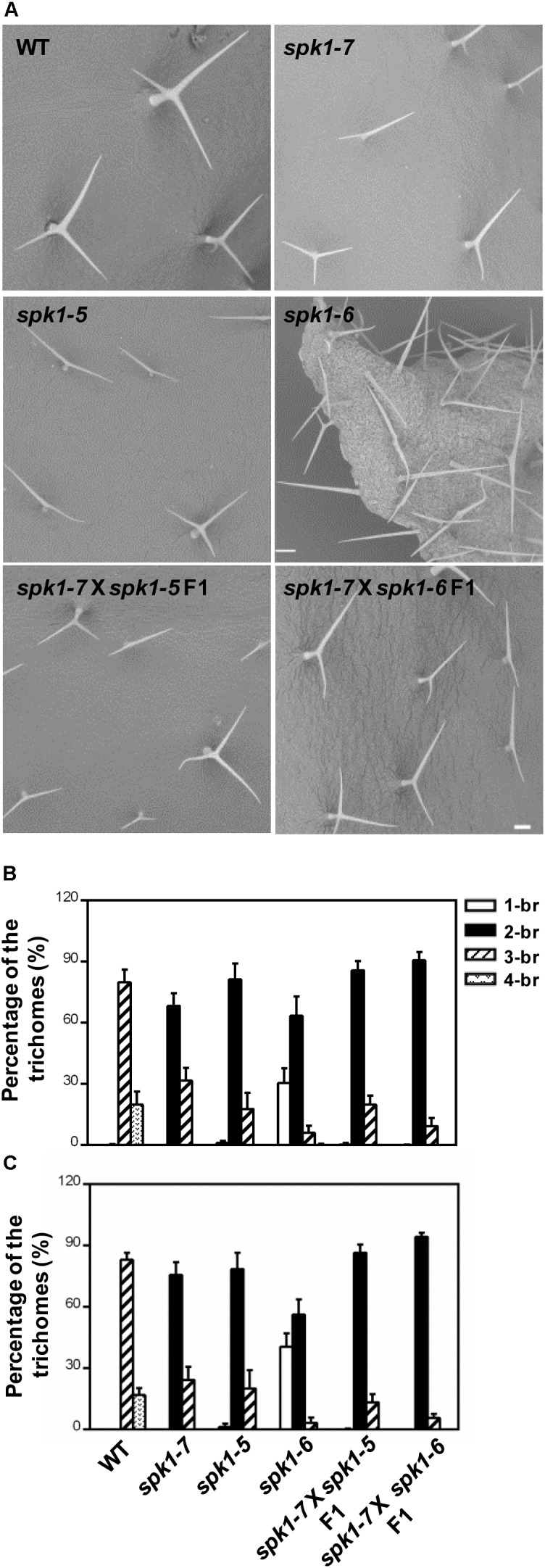
*abt1-1* (*spk1-7*), *spk1-5*, and *spk1-6* are allelic to one another. **(A)** Trichome branching phenotypes on the fifth rosette leaves of WT, *spk1-7, spk1-5, spk1-6, spk1-7* × *spk1-5* F1, and *spk1-7* × *spk1-6* F1 examined by SEM. Bars are 250 μM. **(B,C)** Quantitative analysis of the trichome branching phenotypes on the third **(B)** and fifth **(C)** rosette leaves of indicated genotypes. Data were presented as mean ± SD of three biological replicates. 1/2/3/4-br represented one/two/three/four-branched trichomes, respectively.

Next, we carried out genetic crosses to further assess whether *abt1-1* is allelic to *SALK_136776C* and *SAIL_520H04*. To this end, *abt1-1* was crossed with *SALK_136776C* and *SAIL_520H04*, respectively, and trichome phenotypes of the F1 progeny were examined. While wild type plants scarcely show two-branched trichomes, two-branched trichomes were the most prevalent type found on rosette leaves of the F1 progeny from both crosses (Figures 4A–C). On the third rosette leaves, F1 plants from the cross between *abt1-1* and *SALK_136776C* showed an average of 85.8% two-branched trichomes, while those from the cross between *abt1-1* and *SAIL_520H04* showed an average of 90.6% two-branched trichomes (Figure 4B). Moreover, an average of 13.9% and 9.3% three-branched trichomes were observed in the *abt1-1 SALK_136776C* F1 progeny and the *abt1-1 SAIL_520H04* F1 progeny, respectively, in contrast to the 79.8% in wild type on the fifth rosette leaves (Figure 4C). These observations indicate that *abt1-1* failed to complement *SALK_136776C* or *SAIL_520H04*. *ABT1* and *SPK1* likely represent the same genetic locus. In addition, these data showed that the reduction of trichome branching was more severe in the F1 plants from the cross between *abt1-1* and *SAIL_520H04*, than from the cross between *abt1-1* and *SALK_136776C*, agreeing with the severity of trichome phenotypes of individual alleles. Taken together, we conclude that *ABT1* is *SPK1*, and *abt1-1* is a new mutant allele of *SPK1*. Hence, we designated *SALK_136776C* as *spk1-5, SAIL_520H04* as *spk1-6*, and *abt1-1* as *spk1-7* respectively, according to the literatures.

### The Differential Regulation of Multiple Developmental Processes by *SPK1*

**FIGURE 5:**
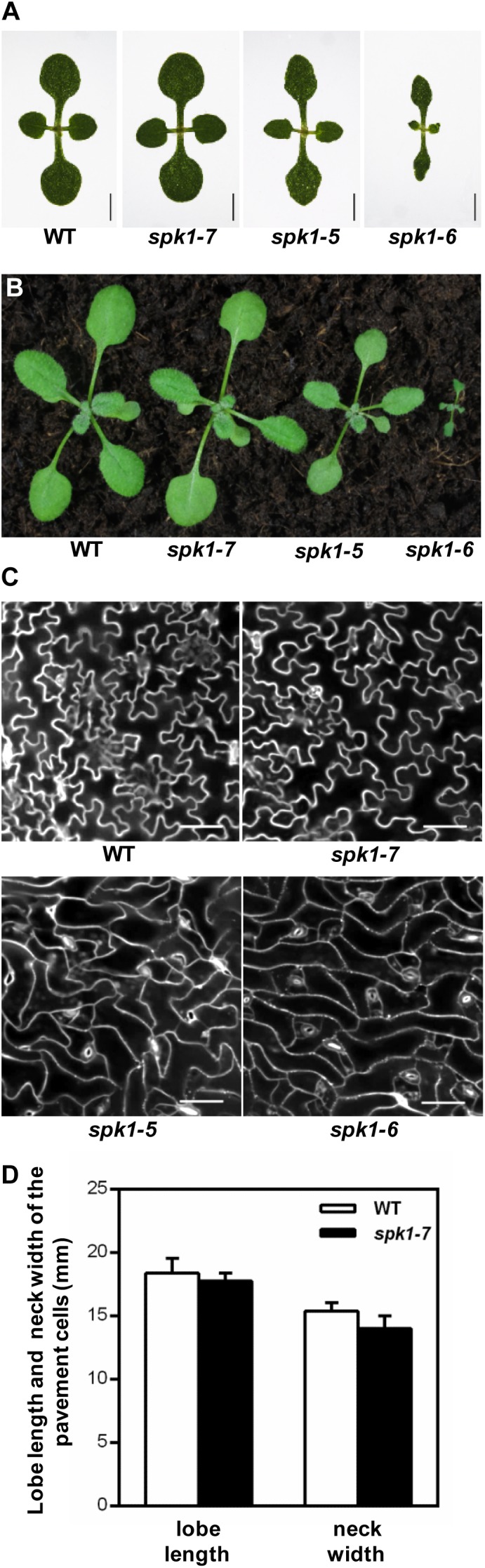
Three different *SPK1* alleles showed varied developmental phenotypes. **(A,B)** Overall plant stature of WT, *spk1-7, spk1-5*, and *spk1-6* at one-week-old **(A)** and two-week-old **(B)**. **(C)** Cotyledon pavement cells morphology of WT, *spk1-7, spk1-5*, and *spk1-6*. The outlines of pavement cells were stained by PI and examined with confocal microcopy. Bars are 20 μM. **(D)** Quantification of the lobe length and the neck width of the cotyledon pavement cell of WT plant and *spk1-7* mutant.

*SPK1* has been shown to regulate several aspects of plant growth and development processes including the overall plant stature, trichome and epidermal pavement cell morphogenesis, as well as leaf, petal and root development (Qiu et al., 2002; Basu et al., 2008; Zhang et al., 2010, 2013; Lin et al., 2012; Ren et al., 2016). Quantification of the trichome branching phenotypes of three *SPK1* alleles clearly showed that different mutations in *SPK1* gene confer varied degree of trichome branching reduction (Figures 3B–D). To explore the potential differential regulation of these processes by *SPK1*, we examined the other two processes regulated by *SPK1*. First, we compared the overall plant stature and found that *spk1-7* resembled the wild type at both seedling and mature stages (Figures 1A, 5A,B). *spk1-5* showed a modest reduction of plant stature and the cotyledon development was also slightly affected (Figures 5A,B). Similar to previously reported strong alleles of *SPK1* (Qiu et al., 2002; Basu et al., 2008), *spk1-6* showed the most severe developmental defects, with small and narrow cotyledons, a gross reduction of plant size and was unable to reach reproductive stage when grown on the soil (Figures 5A,B).

Next, we examined the cotyledon pavement cell morphology in three *SPK1* mutant alleles. In wild type, cotyledon epidermal pavement cells developed pronounced lobes and indentations, showing a characteristic “jigsaw puzzle” arrangement (Figure 5C). In line with other previously reported *spk1* alleles, the outgrowth of pavement cell lobes was greatly repressed and the interdigitation of pavement cells was almost lost in *spk1-5* and *spk1-6* (Figure 5C). Surprisingly, the “jigsaw puzzle” arrangement of pavement cell in *spk1-7* resembled those of the wild type (Figure 5C), and further statistics on the length of lobes and the width of necks of the pavement cells suggested that *spk1-7* mutation didn’t affect pavement cells morphogenesis (Figure 5D). These results indicate that different *SPK1* alleles can lead to differential displays of mutant phenotypes, suggesting that these developmental processes show distinct responses to the activities of *SPK1*in different mutant backgrounds.

### Mutations of *SPK1* Affect Nuclei Positioning in Trichome Cells

The numbers of trichome branches are closely correlated with nuclear DNA content (Tominaga-Wada et al., 2011; Yang and Ye, 2013). Typically, elevated nuclear DNA contents are associated with more trichome branches, while reduced DNA ploidy results in fewer trichome branches. The reduced trichome branching phenotype of *spk1-7* prompted us to test whether *SPK1* is involved in the regulation of endoreduplication and further nuclear DNA ploidy. To this end, we firstly checked the transcript levels of cell cycle related genes in wild type and *spk1-7* mutant alleles. These genes include *CYCD3;1* for the G1 phase; *CDC6a, CDC6b, CDT1a, CDT1b, HISH4*, and *CYCA3;1* for the S phase; *CDKB1;1, CYCB1;1, KRP1*, and *KRP2* for the G2/M phase (Sonoda et al., 2009). No significant differences were observed for the expression levels of these genes in the *spk1-7* mutant compared to those of the wild type (Supplementary Figure S1). Then, we examined the trichome DNA content based on the quantitative fluorescence measurements of DAPI stained nuclei, and found that the relative nuclear DNA content of *spk1-7* trichomes were not remarkably changed compared with that in the wild type (Supplementary Figure S1). These results indicate that reduced trichome branching in the *spk1-7* might not be accompanied with the changes in the trichome nuclear DNA content. *SPK1* may promote trichome branching via a cell cycle independent pathway.

**FIGURE 6:**
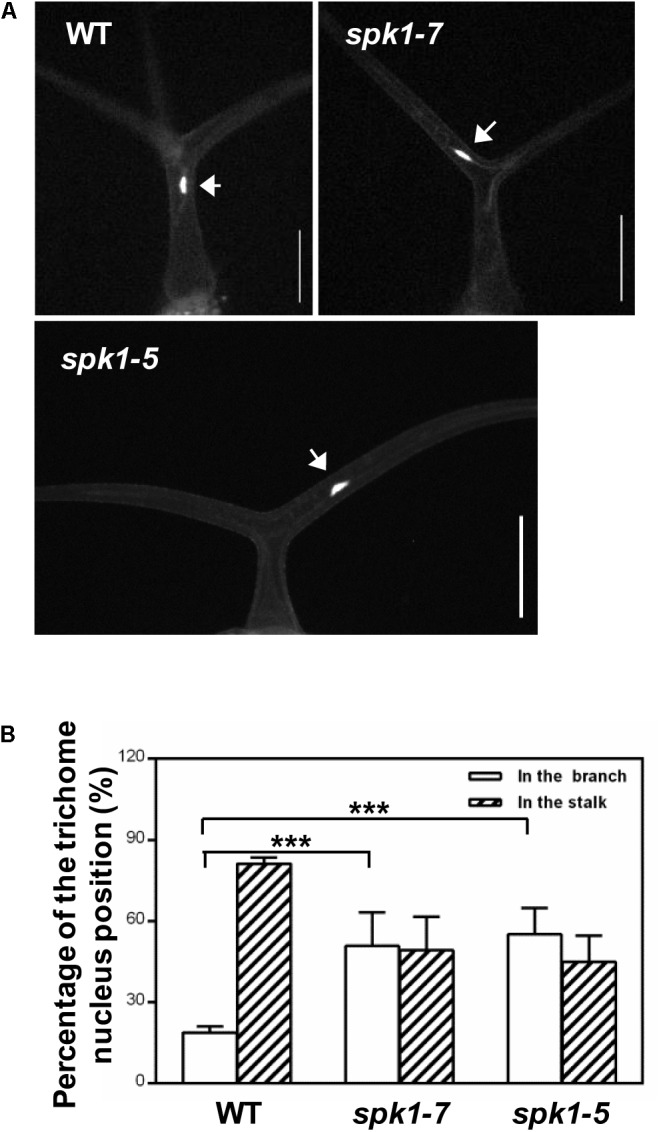
Analysis of trichome nuclei positions in WT and *SPK1* mutants.**(A)** Representative leaf trichomes of WT, *spk1-7* and *spk1-5*. White arrows point to the DAPI-stained trichome nuclei. Images were captured by fluorescence microscopy. Bars are 100 μM. **(B)** Quantification of trichome nuclei positions in WT, *spk1-7* and *spk1-5*. Data were presented as mean ± SD of three biological replicates. ^∗∗∗^*p* < 0.001.

Coincidentally, during the investigation of plant nuclei DNA contents, we observed a high frequency of the abnormally positioned nuclei in two branched trichomes in *spk1-5* and *spk1-7* mutants (Figure 6A). In wild type plants, trichome nuclei were often situated just below the primary branch point in the trichome stalk (Folkers et al., 1997; Figures 6A,B). However, the nuclei of the *spk1-5* and *spk1-7* mutants trichome cells were frequently found in the trichome branches (Figures 6A,B). For the three branched trichomes in *spk1-5* and *spk1-7*, the nuclei position of which was similar to that in the wild type plants (Supplementary Figure S2). In addition, we also examined the nuclei location of the epidermal pavement cells in *spk1-5* and *spk1-7* mutants respectively, and found that there were no remarkable differences among that in wild type and in mutants background. All of the nuclei of the pavement cells examined were resided closed to the cell edges (Supplementary Figure S3). The migration of nuclei during trichome development is a well-known phenomenon but the molecular mechanism underlining this process is poorly understood (Hülskamp et al., 1994; Folkers et al., 1997; Griffis et al., 2014). Our results suggest that *SPK1* might contribute to the correct positioning of trichome nuclei.

### Genetic Interactions Between *SPK1, AN* and *ZWI*

**FIGURE 7:**
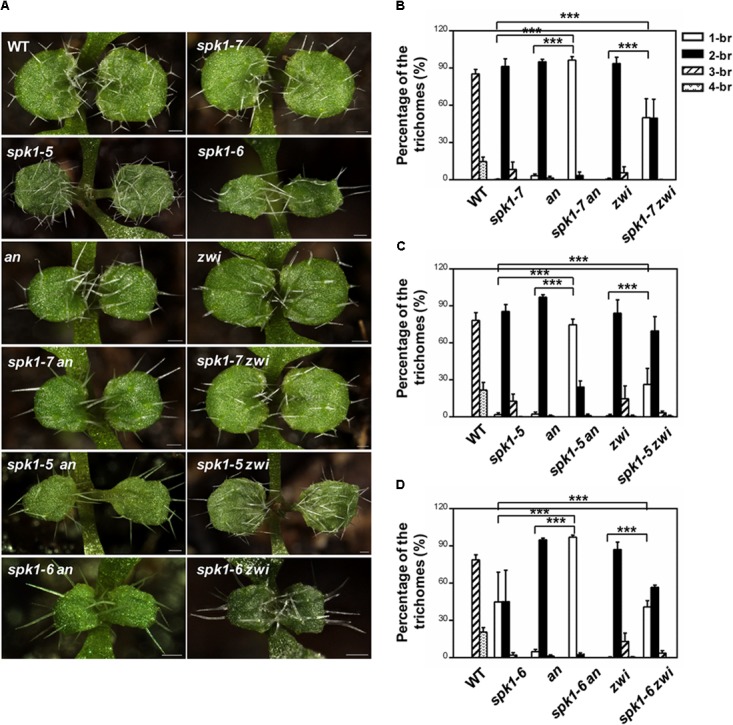
Analysis of the trichome branching phenotype of the double mutants. **(A)** Trichome branching phenotypes on the first pair of rosette leaves of WT, *spk1-7, spk1-5, spk1-6, an, zwi, spk1-7 an, spk1-7 zwi, spk1-5an, spk1-5 zwi, spk1-6 an*, and *spk1-6 zwi double mutants*. Bars are 100 μM. **(B–D)** Quantitative analysis of the trichome branching phenotypes on the third rosette leaves of indicated genotypes. Data were presented as mean ± SD of three biological replicates. ^∗∗∗^*p* < 0.001. 1/2/3/4-br represented one/ two/ three/ four -branched trichomes, respectively.

It is well-known that the migration of the nuclei in the plants cells is tightly correlated with the cMTs status. The abnormal nuclei positions of trichome cells in the *spk1-5* and *spk1-7* mutants reminded us to speculate whether the regulation of trichome branching of *SPK1* depend on the regulation of arrangement of the cMTs cytoskeleton. To investigate this possibility, we tested genetic interactions between *SPK1* and *AN* and *ZWI*, which are characterized to define trichome branching through affecting cMTs dynamics (Reddy et al., 1996; Oppenheimer et al., 1997; Folkers et al., 2002; Kim et al., 2002; Tian et al., 2015). Moreover, loss of functions of either *AN* or *ZWI* displays two-branched trichome phenotypes. We obtained and confirmed T-DNA insertion mutants for *an* (*SALK_026489*) and *zwi* (*SALK_017886*) and generated *spk1-7 an, spk1-7 zwi* double mutants, and examined the trichome branching phenotypes of the double mutants, respectively. In contrast to the predominantly two-branched trichomes in *spk1-7* and *an* single mutant, almost all the trichomes (97.3%) in *spk1-7 an* double mutants only had a single branch (Figures 7A,B). In *spk1-7 zwi* double mutants, although not as dramatic as that in the *spk1-7 an* double mutants, a significant increase in the percentage of single-branched trichomes (54.3%) was observed compared to either of the single mutants (Figures 7A,B). These data implicate that *SPK1* might act coordinately with *AN* and *ZWI* to promote trichome branching. To validate this hypothesis, we also generated double mutants of *spk1-5 an, spk1-5 zwi, spk1-6 an*, and *spk1-6 zwi*, and detected the trichome phenotypes, respectively. Similar to the trichome phenotypes that we observed in *spk1-7 an* double mutants, about 74.5% of the trichomes on the fifth rosette leaves displayed one branched in *spk1-5 an* double mutants (Figures 7A,C). In *spk1-5 zwi* double mutants, although not as conspicuous as that in the *spk1-5 an* double mutants, the number of the one branched trichomes are also remarkably increased (Figures 7A,C). The statuses of trichome phenotypes of the *spk1-6 an* and *spk1-6 zwi* double mutants were the same as those in *spk1-7 an* and *spk1-7 zwi* double mutants, respectively, the trichome branching events were largely prevented (Figures 7A,D).

**FIGURE 8:**
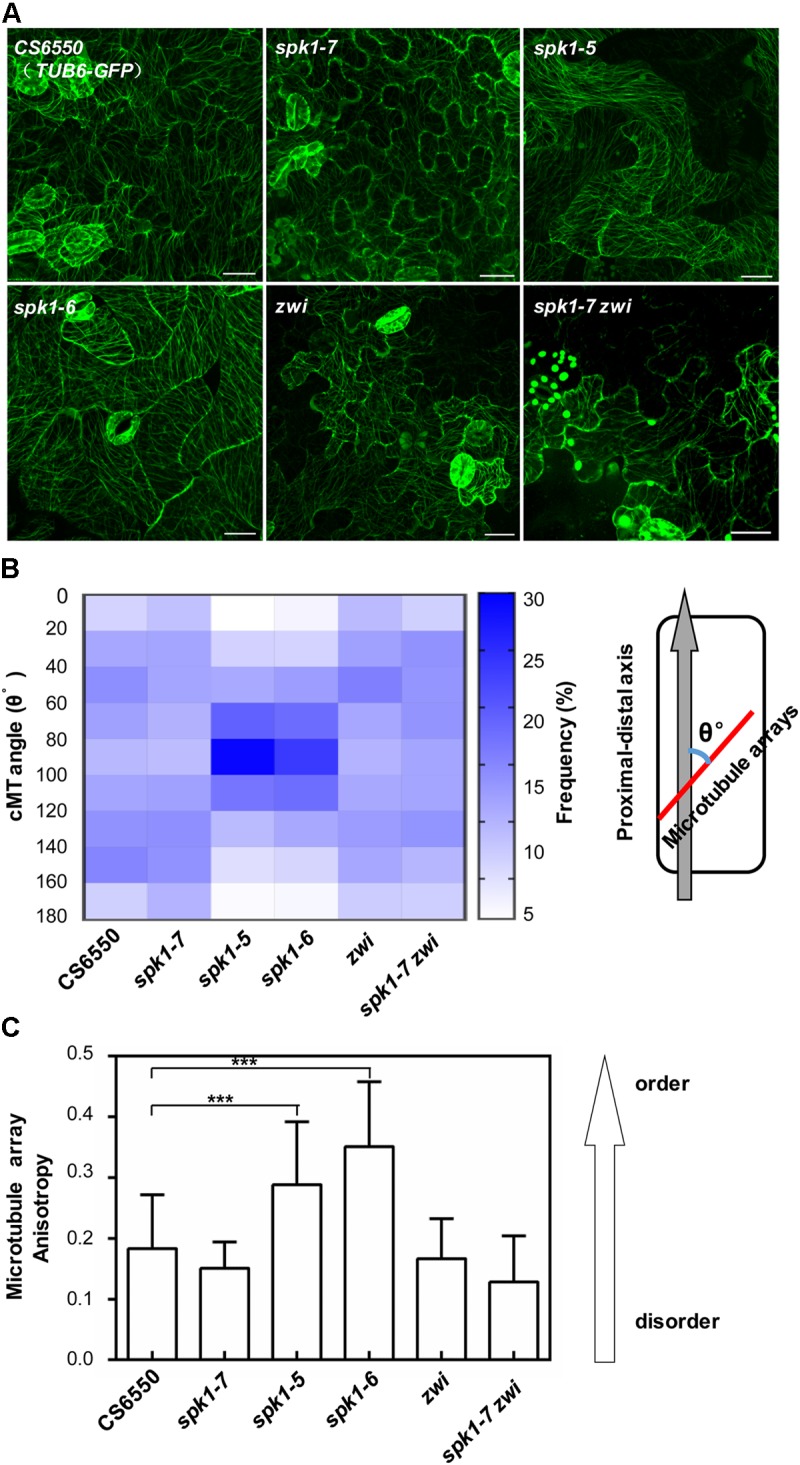
Arrangements of microtubule arrays in the cotyledon pavement cells of *spk1, zwi*, and *spk1-7zwi* double mutants. **(A)** Images of the microtubule arrays in the epidermal pavement cells of 4-day-old cotyledons of the indicated genotypes. Bars are 20 μM for all images. **(B,C)** Quantitative analysis of the microtubule arrays organization by measurement of cMTs angles **(B)** and evaluating the cMTs anisotropy **(C)**. The value of microtubules angles of that parallel to the cell’s longitudinal axis were defined as 0°, while the value of those were perpendicular to the cell’s longitudinal axis were defined as 90°. Approximate 1000 microtubule angles were measured for each genotype, and then were divided into nine intervals (0–20°, 20–40°, 40–60°, 60–80°, 80–100°, 100–120°, 120–140°, 140–160°, 160–180°), respectively. The frequency of the microtubule angles in each interval was analyzed, and finally exhibited as a heatmap. For microtubule anisotropy evaluation, about 10 independent cells for each genotype were used. According to Boudaoud et al. (2014), the anisotropy score zero was defined for no order (purely isotropic arrays) and one for perfectly ordered (purely anisotropic arrays). Data were presented as mean ± SD of three biological replicates. ^∗∗∗^*p* < 0.001.

Considering the genetic interaction among *AN, ZWI*, and *SPK1*, we were interested in further understanding whether the regulation of epidermal pavement cell development by *SPK1* is also associated with the cMTs arrays dynamics. We detected the cMTs arrangements in the pavement cells of cotyledons of *spk1-5, spk1-6*, and *spk1-7* mutants, respectively. As shown in Figure 8A, the configuration of cMTs arrays in *spk1-7* mutant was observed to similar to that in the wild type (*CS6550*), but in the *spk1-5* and *spk1-6* background, the cMTs arrays were assembled more orderly, which was largely different from that in the wild type, suggesting a certain degree of correlation between the pavement cell shape and the cMTs arrangements. The regulation of *SPK1* on the pavement cell development might due to its influences on the cMTs dynamics. To validate this point, we measured the cMTs angles and quantitatively analyzed their distribution. In consistent with our observation, the quantitative data showed in wild type and *spk1-7* mutant, the cMTs angles displayed a relative even distribution in different orientations, while in the *spk1-5* and *spk1-6* mutants, a significant peak of cMTs angles were observed between 80°-100° (Figure 8B). We also examined the anisotropy of the cMTs in wild type, *spk1-7, spk1-5* and *spk1-6* mutants, and found that the mean of the anisotropy value in *spk1-5* and *spk1-6* mutants were about 0.29 ± 0.10 and 0.35 ± 0.11, respectively, compared to that of about 0.18 ± 0.09 in wild type and 0.15 ± 0.04 in *spk1-7* mutant (Figure 8C), implicating the isotropic arrangements of cMTs in *spk1-5* and *spk1-6* mutants comparing to that in wild type plant. We detected the organization of the cMT arrays in the pavement cells in the *spk1-7 zwi* double mutants background at the same time, and found that the status of the cMTs arrays resembled to that in *zwi* single mutants (Figures 8A–C). These data indicate that *SPK1* might affect cMTs cytoskeleton dynamics through *AN* and *ZWI* during plant development.

**FIGURE 9:**
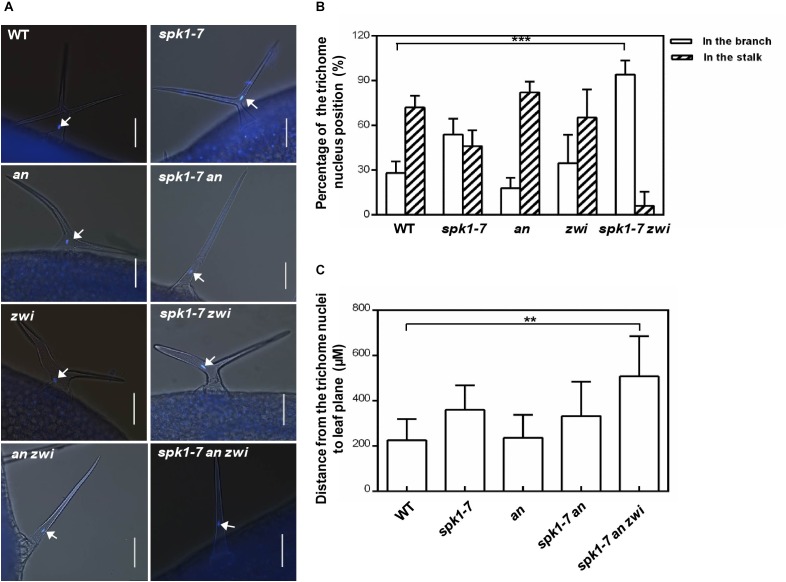
Nuclei positioning statues of the trichome cells in different mutants. **(A)** Representative trichomes images of the indicated genotype plants. White arrows point to the DAPI-stained trichome nuclei. Bars are 100 μM. **(B)** Quantification of trichome nuclei positions in WT, *spk1-7, an, zwi*, and *spk1-7 zwi* double mutants. **(C)** Statistic analysis of the distance between the trichome nulei and the leaf plane in WT, *an, spk1-7an double mutants*, and *spk1-7an zwi* triple mutants. Data were presented as mean ± SD. ^∗∗∗^*p* < 0.001, ^∗∗^*p* < 0.005.

To further analysis the interactions among *SPK1* and *AN* and *ZWI* during plant development, we also investigated the nuclei positions of trichome cells in the *spk1-7 an* and *spk1-7 zwi* double mutants, respectively. In *an* and *zwi* single mutant, a large portion of the trichome nuclei resided in the stalk, just below the primary branch point (Figures 9A,B), which was similar to that in the wild type, while in *spk1-7zwi* double mutants about 93.7% of the trichome nuclei were positioned at the branches (Figures 9A,B). When we looked at the trichome branching phenotypes in the *spk1-7an* double mutants and *spk1-7 an zwi* triple mutants, found almost all of the trichomes are single-branched (Figure 9A), so we measured the distance between the nuclei and the leaf plane. The data showed that the trichome nuclei were remarkably migrated far away from the leaf plane based on our quantitative analysis (Figure 9C). These data indicate that *SPK1* might contribute to the regulation of the migration of the trichome nuclei, while *AN* and *ZWI* tend to serve for the cMTs arrays dynamic during trichome branching, and they work together to define the precise trichome branching events.

## Discussion

### The Identification of a Novel Allele of *SPK1*

The differentiation and specification of defined cell types is a hallmark of the development of multi-cellular organisms. In higher plants, trichome cells offer an excellent paradigm for cell differentiation and morphogenesis because of their delicate growth patterns (Hülskamp et al., 1994). The long-term goal of our group is to unravel the molecular mechanisms of trichome cell differentiation, and we have carried out large-scale genetic screens in *A. thaliana* for mutants with abnormal trichome morphology. In this work, we report the isolation and identification of a novel trichome branching mutant *abt1-1*. Molecular cloning and genetic analysis revealed that *ABT1* is identical to the previously reported locus *SPK1* and thus our mutant allele represents a novel allele of *SPK1* (Figures 2–4). Therefore, *abt1-1* was renamed as *spk1-7* according to the previously reported alleles.

In Arabidopsis, previous findings clearly indicate that *SPK1* is indispensable for plant development and influences a spectrum of developmental programs including at least three well-defined processes: the overall plant stature, epidermal pavement cell and trichome cell morphogenesis (Qiu et al., 2002). However, further functional and genetic characterizations of *SPK1* have been hampered by the seedling lethality of null *spk1* mutants. In this work, interestingly, we isolated a fully viable hypomorphic allele of *SPK1, spk1-7*, which can serve as a valuable tool for future characterizations of *SPK1* functions. In contrast to the previously reported seedling lethality of null *spk1* mutants, the growth status including the overall plant stature, as well as pavement cell morphology of *spk1-7* is not different from that of wild type plants except for the two branched trichomes (Figure 1). Molecular data showed a single G to A transition mutation at the +5893 position (from the ATG start codon) in the *SPK1* locus in *spk1-7* mutant background (Figure 2). According to the predicted *SPK1* amino acid sequences, this mutation would convert a glycine codon (GGA, Gly760) to a codon for glutamate (GAA, Glu760). *SPK1* is a large protein with several conserved domains: the pivotal C-terminal CDM domain, other two domains named DHR3/MOD1 and DHR1/MOD2, respectively (Qiu et al., 2002; Basu et al., 2008). The CDM domain has been characterized to confer the *SPK1* functioning as a GEF. In this pathway, *SPK1* promotes the activities of the conserved Rho-of-Plants (ROP) GTPases by facilitating the exchange of GDP to GTP (Côté and Vuori, 2007; Basu et al., 2008). In turn, activated ROP GTPases regulate the actin cytoskeleton through the SCAR/WAVE-ARP2/3 complexes (Côté and Vuori, 2007; Basu et al., 2008). However, the function of other two domains DHR3/MOD1 and DHR1/MOD2 are still unclear. The mutation site of *spk1-7* locates in the middle of the protein, neither in the DHR3/MOD1 nor DHR1/MOD2. This sense mutation doesn’t affect the accumulation of the transcripts of *SPK1* in *spk1-7* mutant (Figure 3E), and cause only the trichome development impairment, suggesting that this region might specifically interact with the trichome development regulators. The conversion of the glycine codon to the glutamate codon might influence the physical interactions among *SPK1* and other factors.

### The Differential Regulation of Overall Plant Stature, Epidermal Pavement Cell and Trichome Cell Differentiation by *SPK1*

*SPK1* has been reported to play essential roles in plant growth and development and null alleles of *SPK1* display gross alterations of plant development with dwarfed stature, narrow and distorted cotyledons and rosette leaves, leaf trichome and pavement cell defects, eventually leading to seedling lethality (Qiu et al., 2002). In this work, taking advantage of the three *SPK1* mutant alleles of varied severity (Figures 3, 5), we were able to probe the differential regulation of the above-mentioned three developmental processes by *SPK1*. Based on our results, trichome cell development appears to be the most sensitive to genetic disturbance of *SPK1*. In *spk1-7* mutant, the overall plant development was not visibly affected, yet trichome cell branching was clearly impacted by the *spk1-7* mutation (Figure 1). The further reduction of *SPK1* activities appears to affect the trichome cell branching and pavement cell morphogenesis more (Figure 3E). As in *spk1-5*, the pavement cell development was grossly altered, similar to the strongest allele *spk1-6* and other reported null mutants of *SPK1*, yet the overall growth and development was only slightly altered (Figures 5A,B). In strong allele such as *spk1-6*, all three developmental processes were impacted, similar to previous null mutants (Figures 3, 5; Qiu et al., 2002). *spk1-5* and *spk1-6* are all T-DNA insertional alleles of *SPK1*, and the T-DNAs were inserted in the 27th intron and the in the ninth exon of the *SPK1* gene in *spk1-5* and *spk1-6*, respectively. These two regions represent the DHR2 domain and the DHR3 domain, respectively, suggesting these two domains might involve in other development processes regulation besides trichome branching control. Moreover, the regulation of *SPK1* on cotyledon pavement cell development and trichome branching might refer to cMTs dynamics, as in *spk1-5* and *spk1-6* mutants which exhibited conspicuous cotyledon pavement cell development impact, the pavement cell cMTs were arranged more orderly, about 25%-30% cMTs oriented to be perpendicular to the cell’s longitudinal axis (Figures 8A–C). In *spk1-7* mutant, the pavement cell morphology was undistinguished from that of wild type plants, in consistent, the cMTs organization was similar to that in wild type plants, and the orientation of cMTs was randomly (Figures 8A–C). However, in two-branched trichomes, we found that the distribution of the cMTs were relative isotropic compared that in wild type (Supplementary Figure S4). Taken together, our results raise several interesting scenarios regarding the mode of operation by *SPK1*. It is possible that different developmental processes may have differential responses to the overall level *SPK1* activities. Alternatively, different domains of *SPK1*may confer differential functions in different developmental processes.

### The Genetic Interactions Between *SPK1* and *AN* and *ZWI*

The discovery of the regulation of the nuclei positioning of trichome cell by *SPK1* promoted us to probe how *SPK1* interacts genetically with *AN* and *ZWI*, two other known regulators of trichome branching determination. Loss of function of *AN* results in plants with narrow leaves, two branched trichomes and pavement cell shape defects (Tsuge et al., 1996). *AN* encodes a plant homolog of the C-terminal binding proteins/brefeldin A ADP-ribosylated substrates (CtBP/BARS) protein (Folkers et al., 2002; Kim et al., 2002). In *an* mutant, the arrangement of cMTs cytoskeleton is disturbed and the abnormal cMTs is thought to cause the mutant phenotypes (Folkers et al., 2002; Kim et al., 2002). Although originally thought to be localized to the nucleus and function as a transcriptional co-repressor, more recent data suggest that *AN* may partially localize to the *trans*-Golgi network (TGN) and may exert its functions through the endomembrane system (Minamisawa et al., 2011). In *spk1-7 an* double mutants, the majority of trichomes are single branched (Figures 7A,B), suggesting that *SPK1* and *AN* interact genetically in regulating trichome branch formation. Considering that *SPK1* is associated with ER, it t is likely that endomembrane system including ER and TGN may be involved in the cMTs organization and trichome morphogenesis. Our findings with the double mutant of *spk1-7* and *zwi* further support a functional link between *SPK1* and the organization of cMTs. In contrast to *an, zwi* mutant also shows two branched trichomes with short stalks, but the overall plant growth and development is not significantly affected (Hülskamp et al., 1994). *ZWI* encodes a plant microtubule associated protein (MAP) that is known as kinesin-like calmodulin-binding protein (KCBP) (Reddy et al., 1996; Oppenheimer et al., 1997; Abdel-Ghany et al., 2005), and could bind to the microtubules (Narasimhulu and Reddy, 1998). *spk1-7 zwi* double mutants showed increased percentage of single-branched trichomes that are similar to those of *spk1-7 an* double mutants (Figures 7A,B). Interestingly ZWI/KCBP has been reported to physically interact with *AN* (Folkers et al., 2002). The similar phenotypes we observed in *spk1-7 an* and *spk1-7 zwi* double mutants are consistent with this notion. Moreover, we found that unlike that in *an* mutant, the gross pavement cell morphology and cMTs organization in *zwi* as well as in *spk1-7 zwi* mutant were similar to that in wild type (Figures 8A–C). These data implicated that *SPK1* might interact with *ZWI* to specifically regulate trichome branching.

Another interesting phenomenon we observed is the aberrant placements of the trichome nuclei in *spk1* mutants (Figures 6A,B). Nucleus is the core of the eukaryotic cell, and its position is not fixed in the cell. It needs to move to a proper position depending on the cell type, developmental process, and physiological situations (Griffis et al., 2014; Tan et al., 2016; Nakamura et al., 2018). In the mature trichome cells, the nuclei usually are positioned in the stalk, just below the primary branch points (Folkers et al., 1997), and this positioning pattern is considered to depend on the dynamic cMTs, either cMT destabilizing or stabilizing could alter the nuclei positions of trichome cells (Folkers et al., 1997; Mathur and Chua, 2000). However, we found that loss of function of *AN* seems to have no remarkable effects on nuclei positioning of the trichome cells. In *an* mutant, most of the nuclei of the trichome cells reside in the stalk (Figures 9A,B), and the distance from the trichome cell nuclei to the leaf plane is about 237.9 μM, which is not different from that in the wild plants (Figure 9C). In the *spk1-7 an* double mutant, although the positions of the nuclei are elevated, it is not remarkable different from that in *spk1-7* plants (Figure 9C). When we investigated the nuclei positions of trichome cells in the *spk1-7 an zwi* triple mutant, we found that the positions of nuclei are about 508 μM far away from the leaf plane (Figure 9C), which is significantly elevated compared that in *spk1-7* plants. These data implicate a coordinative regulation pattern of *SPK1* and *AN* on trichome branching. *SPK1* might interact with *ZWI* to contribute to the regulation of the migration of the trichome nuclei, while *AN* tends to serve for the cMTs arrays dynamic during trichome branching.

Taken together, based on our data, *SPK1, AN* and *ZWI* would all act as positive regulators of trichome branch formation, and their roles in promoting the formation of trichome branches likely involve the microtubule cytoskeleton. It would be interesting in the future to uncover the underlying mechanism of *SPK1* on regulating nuclei migration during trichome branching and the functional relationship between *SPK1* and the microtubule cytoskeleton.

## Author Contributions

LA, FY, and SL designed the experiments. SL, XY, JZ, and JS carried out the experiments. SL and LA wrote the manuscript. XL, YQ, and FY revised the article. MD conducted the additive data in the revised manuscript. All authors agreed to be accountable for the content of the work and gave the final approval for the submission of the manuscript.

## Conflict of Interest Statement

The authors declare that the research was conducted in the absence of any commercial or financial relationships that could be construed as a potential conflict of interest.
