# Supplementary material for: A New Allele of the SPIKE1 Locus Reveals Distinct Regulation of Trichome and Pavement Cell Development and Plant Growth
Source: Front Plant Sci. 2019 Jan 24;10:16. doi: 10.3389/fpls.2019.00016 (PMC6353857; doi:10.3389/fpls.2019.00016)
Supplement: Supplementary file 1 [file Presentation_1.PPTX]

## Slide 1
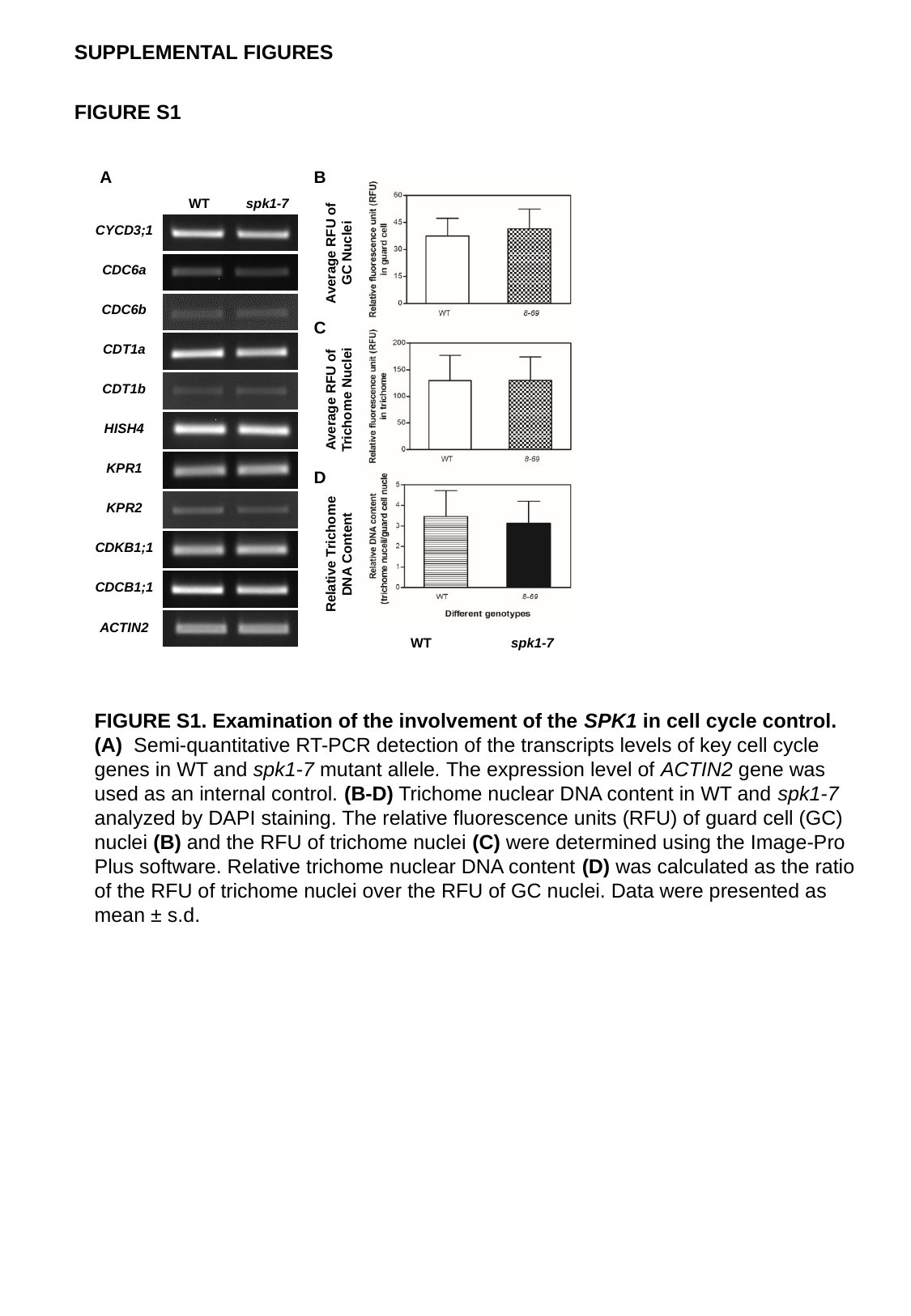

SUPPLEMENTAL FIGURES
FIGURE S1
A
B
spk1-7
 WT
CYCD3;1
Average RFU of
GC Nuclei
CDC6a
CDC6b
C
CDT1a
CDT1b
Average RFU of
Trichome Nuclei
HISH4
KPR1
D
KPR2
Relative Trichome
DNA Content
CDKB1;1
CDCB1;1
ACTIN2
WT
spk1-7
FIGURE S1. Examination of the involvement of the SPK1 in cell cycle control.
(A) Semi-quantitative RT-PCR detection of the transcripts levels of key cell cycle genes in WT and spk1-7 mutant allele. The expression level of ACTIN2 gene was used as an internal control. (B-D) Trichome nuclear DNA content in WT and spk1-7 analyzed by DAPI staining. The relative fluorescence units (RFU) of guard cell (GC) nuclei (B) and the RFU of trichome nuclei (C) were determined using the Image-Pro Plus software. Relative trichome nuclear DNA content (D) was calculated as the ratio of the RFU of trichome nuclei over the RFU of GC nuclei. Data were presented as mean ± s.d.

## Slide 2
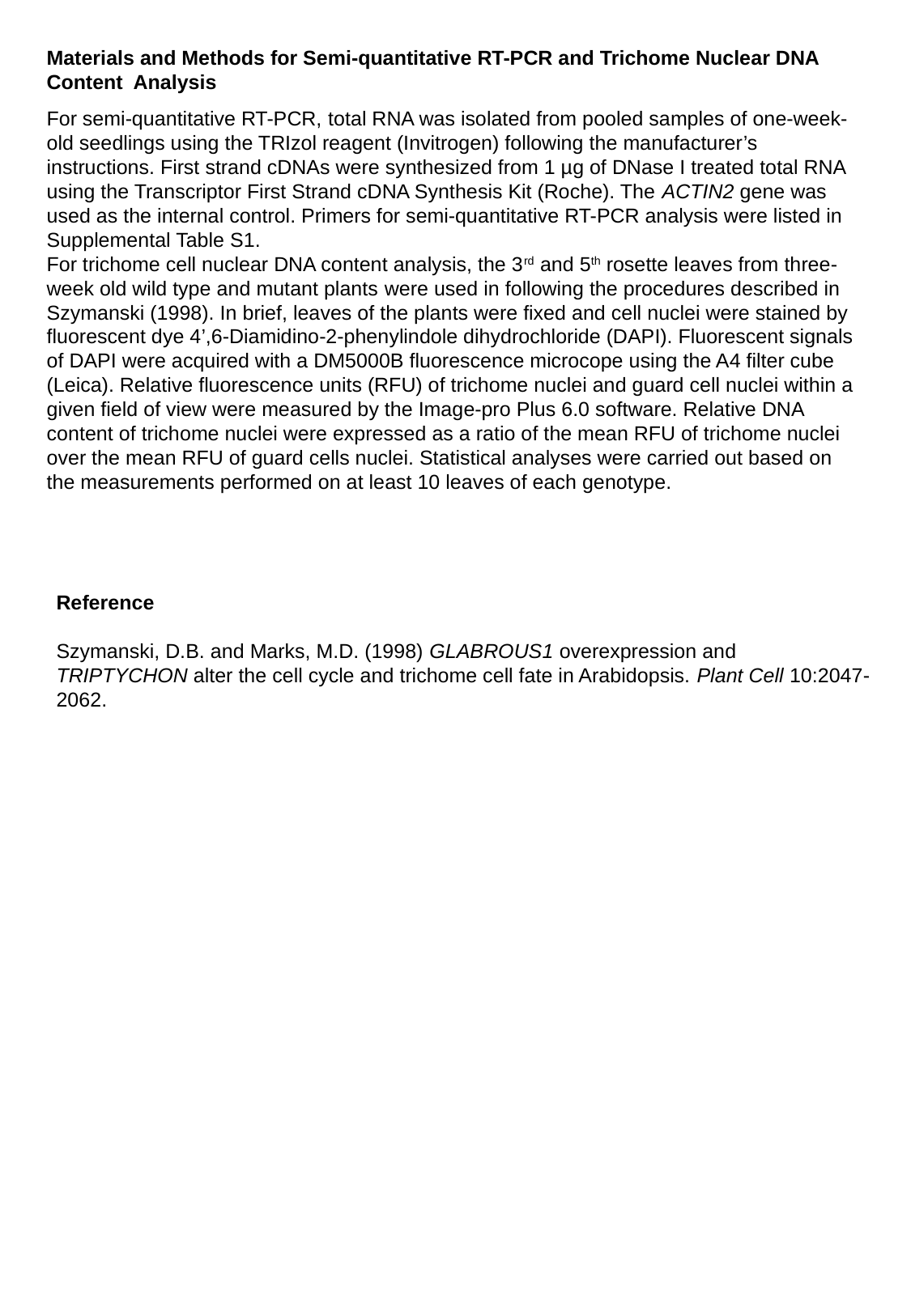

Materials and Methods for Semi-quantitative RT-PCR and Trichome Nuclear DNA Content Analysis
For semi-quantitative RT-PCR, total RNA was isolated from pooled samples of one-week-old seedlings using the TRIzol reagent (Invitrogen) following the manufacturer’s instructions. First strand cDNAs were synthesized from 1 µg of DNase I treated total RNA using the Transcriptor First Strand cDNA Synthesis Kit (Roche). The ACTIN2 gene was used as the internal control. Primers for semi-quantitative RT-PCR analysis were listed in Supplemental Table S1.
For trichome cell nuclear DNA content analysis, the 3rd and 5th rosette leaves from three-week old wild type and mutant plants were used in following the procedures described in Szymanski (1998). In brief, leaves of the plants were fixed and cell nuclei were stained by fluorescent dye 4’,6-Diamidino-2-phenylindole dihydrochloride (DAPI). Fluorescent signals of DAPI were acquired with a DM5000B fluorescence microcope using the A4 filter cube (Leica). Relative fluorescence units (RFU) of trichome nuclei and guard cell nuclei within a given field of view were measured by the Image-pro Plus 6.0 software. Relative DNA content of trichome nuclei were expressed as a ratio of the mean RFU of trichome nuclei over the mean RFU of guard cells nuclei. Statistical analyses were carried out based on the measurements performed on at least 10 leaves of each genotype.
Reference
Szymanski, D.B. and Marks, M.D. (1998) GLABROUS1 overexpression and TRIPTYCHON alter the cell cycle and trichome cell fate in Arabidopsis. Plant Cell 10:2047-2062.

## Slide 3
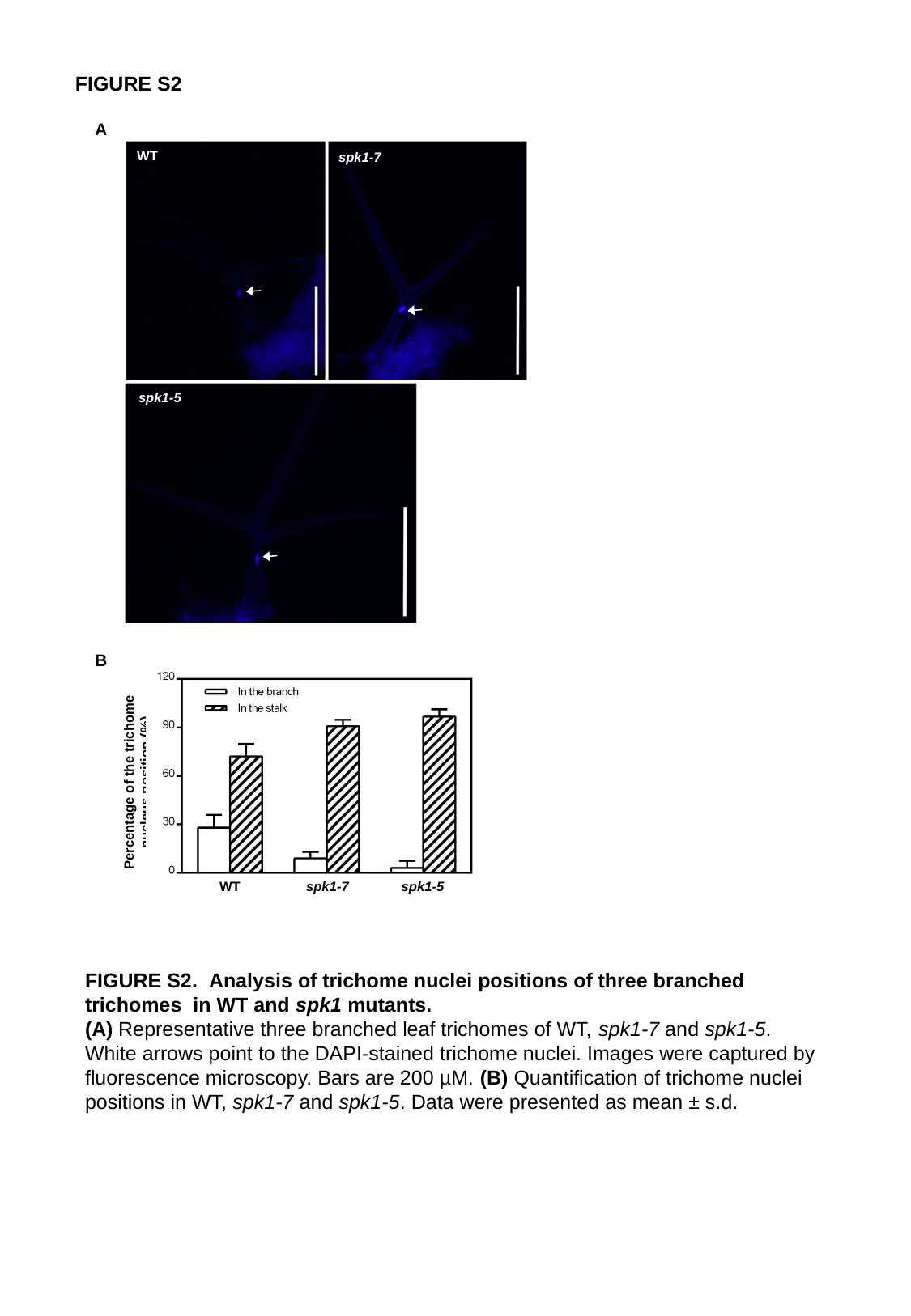

FIGURE S2
A
WT
spk1-7
spk1-5
B
WT
spk1-7
spk1-5
Percentage of the trichome
nucleus position (%)
FIGURE S2. Analysis of trichome nuclei positions of three branched trichomes in WT and spk1 mutants.
(A) Representative three branched leaf trichomes of WT, spk1-7 and spk1-5. White arrows point to the DAPI-stained trichome nuclei. Images were captured by fluorescence microscopy. Bars are 200 µM. (B) Quantification of trichome nuclei positions in WT, spk1-7 and spk1-5. Data were presented as mean ± s.d.

## Slide 4
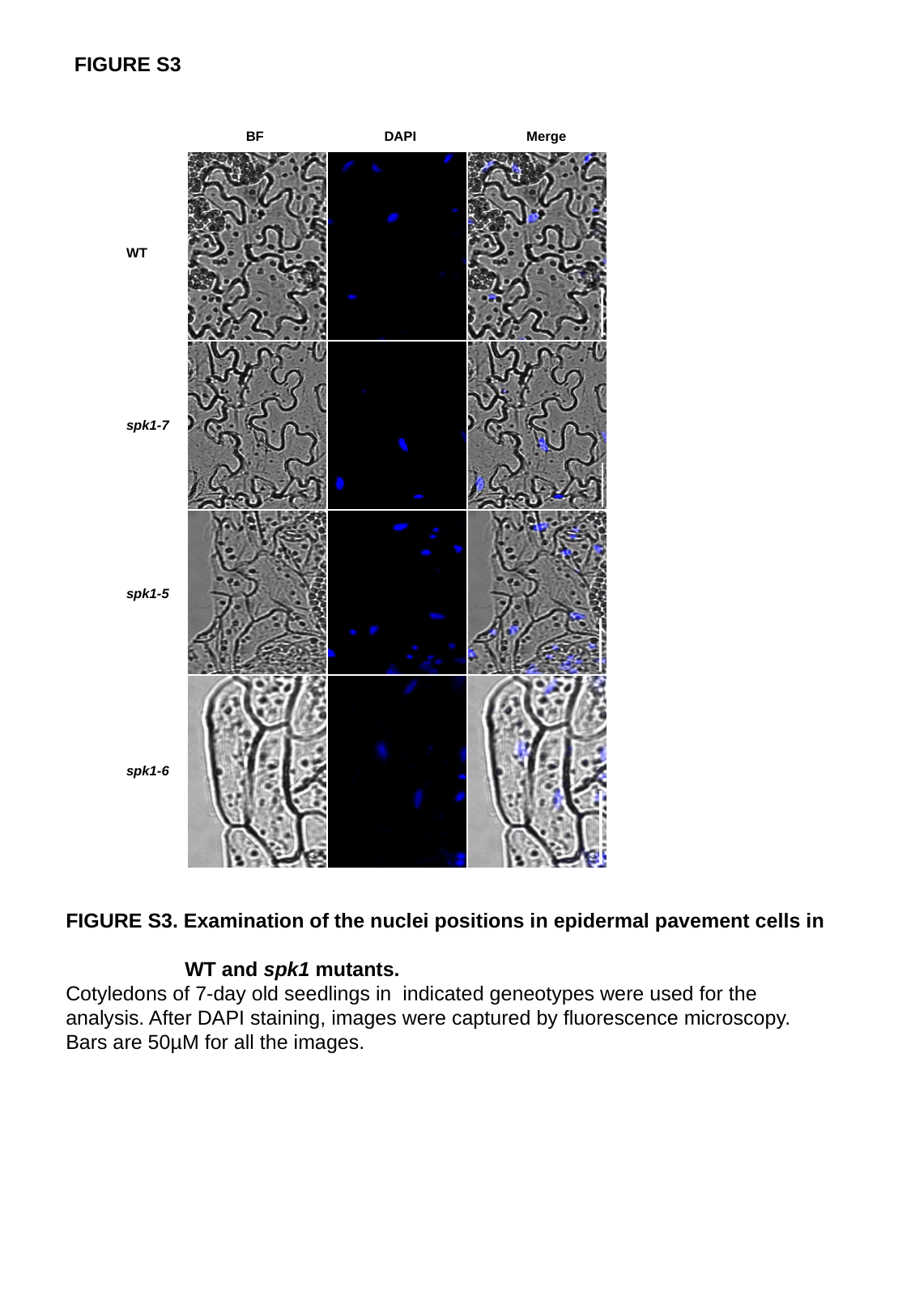

FIGURE S3
BF
DAPI
Merge
WT
spk1-7
spk1-5
spk1-6
FIGURE S3. Examination of the nuclei positions in epidermal pavement cells in
 WT and spk1 mutants.
Cotyledons of 7-day old seedlings in indicated geneotypes were used for the analysis. After DAPI staining, images were captured by fluorescence microscopy.
Bars are 50µM for all the images.

## Slide 5
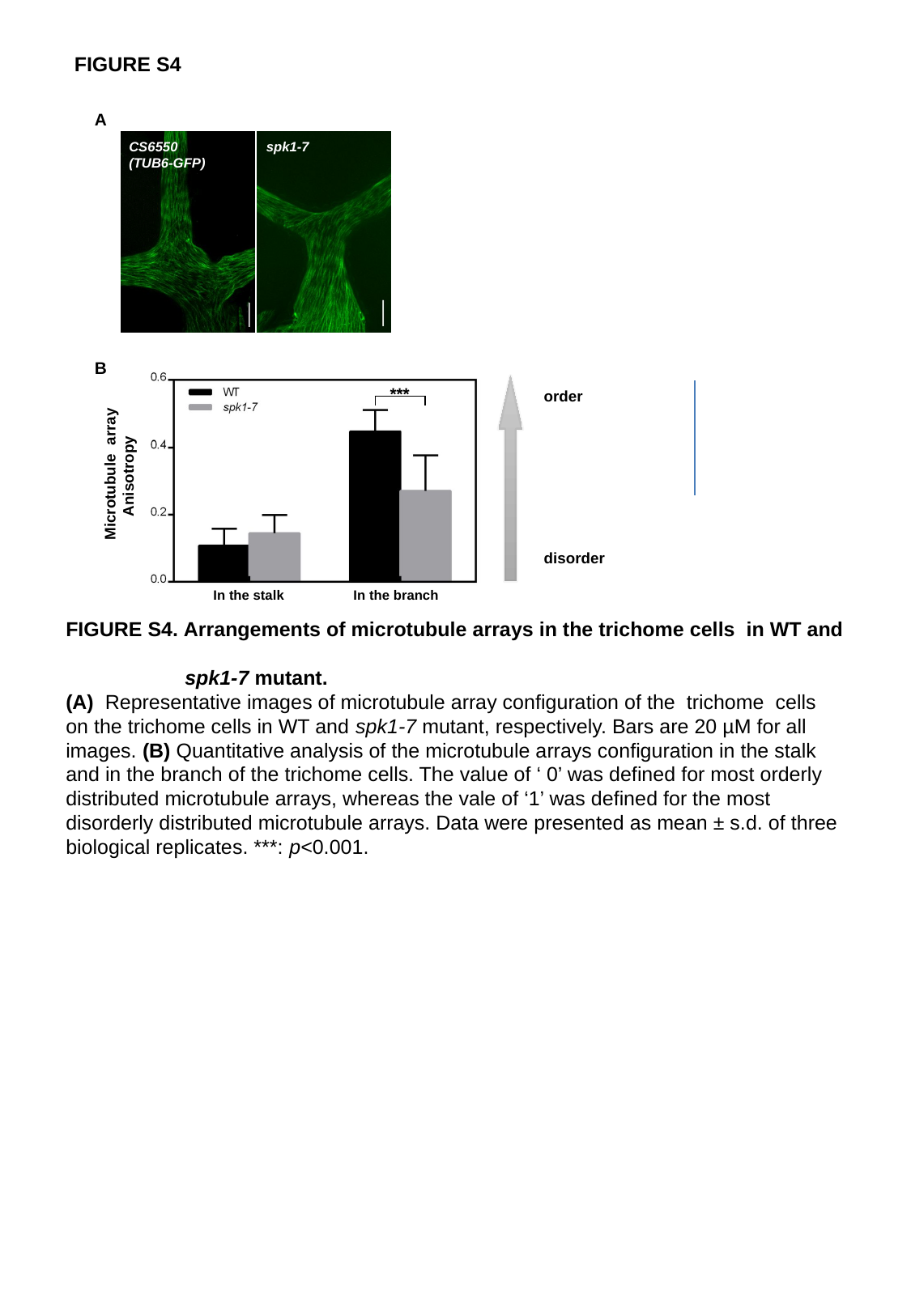

FIGURE S4
A
CS6550
(TUB6-GFP)
spk1-7
B
 Microtubule array Anisotropy
order
disorder
In the stalk
In the branch
***
FIGURE S4. Arrangements of microtubule arrays in the trichome cells in WT and
 spk1-7 mutant.
(A) Representative images of microtubule array configuration of the trichome cells on the trichome cells in WT and spk1-7 mutant, respectively. Bars are 20 µM for all images. (B) Quantitative analysis of the microtubule arrays configuration in the stalk and in the branch of the trichome cells. The value of ‘ 0’ was defined for most orderly distributed microtubule arrays, whereas the vale of ‘1’ was defined for the most disorderly distributed microtubule arrays. Data were presented as mean ± s.d. of three biological replicates. ***: p<0.001.
