# Supplementary material for: A New Allele of the SPIKE1 Locus Reveals Distinct Regulation of Trichome and Pavement Cell Development and Plant Growth
Source: Front Plant Sci. 2019 Jan 24;10:16. doi: 10.3389/fpls.2019.00016 (PMC6353857; doi:10.3389/fpls.2019.00016)
Supplement: Supplementary file 2 [file Table_1.DOC]

Table 1 Primer pairs used in this study.

| Name | Sequence (5´-3´) | Comments |
| --- | --- | --- |
| *abt1-1* LP  *abt1-1* RP  SALK_136776 LP | TGATGATTTTGGAGGCAAT  CATGGTCGGAGATAATTTGC  ACGGAAGGTTCACAAACCTG | For *abt1-1* mutant mutation site sequencing  For homozygous mutant |
| SALK_136776 RP | GTATGAACCCATTTCTGGAG | genotyping |
| SAIL_520H04 LP | GACGATTACACTGTCAGAAC | For homozygous mutant |
| SAIL_520H04 RP | CACATAGATGCAATAACCAC | genotyping |
| SALK_026489 LP | CCATGTAGATACTTCACGAG | For homozygous mutant |
| SALK_026489 RP | ATCTCCATCCATACTTCCTC | genotyping |
| SALK_017886 LP | AAGGTGATGGAGCACTATTG | For homozygous mutant |
| SALK_017886 RP | GGAATTGACAATGCCACTTG | genotyping |
| FCA2#1 LP | CACTTCGTCCTGTAACATAG | For positional cloning |
| FCA2#1 RP | TCCTCCTAGAGTGTTTGTTG |  |
| F17L22#1 LP | TCCCACGGGTTTATCACATC | For positional cloning |
| F17L22#1 RP | CCTTCCTAATGTAAACCGGC | For homozygous mutant  genotyping |
| AN LP | CCATGTAGATACTTCACGAG |
| AN RP | AGCACATCTAGAACTGGAAC |
| ZWI LP | ACTTGCTGAGCTAGAAATAC | For homozygous mutant  genotyping |
| ZWI RP | CTATCTGCCTCATCTTTTCG |
| SPK1 RT-F1 | AGGTCTCTCTTCCTGCCACA | For real-time quantitative RT-PCR |
| SPK1 RT-R1 | CTCTCCTTGGTGCTTTCCTG |
| SPK1 RT-F2 | GTTGGGAAGTTGGACAGGA | For real-time quantitative RT-PCR |
| SPK1 RT-R2 | CCCAAGATCCTCATCTTCCA |
| CYCD3;1 RT-LP | TTGTAGCTCTCCCCTGCTAA | For semi-quantitative RT-PCR |
| CYCD3;1 RT- RP | AGACAGCTGAGTCCTTGTTC |
| CDC6a RT-L | ATTCGGCTGCGGAGGTTTCA | For semi-quantitative RT-PCR |
| CDC6a RT-RP | CCCGGACTCATAGTTACCAA |
| CDC6b RT-LP | ATATTGTAGCCATCGGAACGA | For semi-quantitative RT-PCR |
| CDC6b RT-RP | GAAGAGTTGGTGTTAGCATTC |
| CDT1a RT-LP | GGCGAAAGATGATCGCTTGT | For semi-quantitative RT-PCR |
| CDT1a RT-RP | GGCGAAAGATGATCGCTTGT |
| CDT1b RT-LP | GAGGAAGATGCAATTGAGAAAG | For semi-quantitative RT-PCR |
| CDT1b RT-RP | TCAGTAGATAAGTGAAATGTCAT |
| HISH4 RT-LP | TTCTGAGAGACAACATCCAAGGA | For semi-quantitative RT-PCR |
| HISH4 RT-RP | AAGAGTCCTTCCTTGCCTCTT |
| KRP1 RT-LP | TGAGAGAGGAGGAGAAAGAAG | For semi-quantitative RT-PCR |
| KRP1 RT-RP | CTAACTTTACCCATTCGTAACG |
| KRP2 RT-LP | TCTTCCTCCACCAAGTGGCTC | For semi-quantitative RT-PCR |
| KRP2 RT-RP | TCTTCCTCCACCAAGTGGCTC |
| CDKB1;1 RT-LP | ACGAAACCTCTCAGAAATGG | For semi-quantitative RT-PCR |
| CDKB1;1 RT-RP | CTCAGTTGGTGTTCCTAGCA |
| CYCB1;1 RT-LP | GTGGAGGATAATCTCAAAAAACC | For semi-quantitative RT-PCR |
| CYCB1;1 RT-RP | ACCAGAATCTGTTTGTGACTGTA |
| ACTIN2 RT-LP | TCAAAGACCAGCTCTTCCATCGAGA | Internal control for semi-quantitative RT-PCR |
| ACTIN2 RT-RP | ACACACAAGCGCATCATAGAAACGA |
| ACTIN2 RT-LP | TCAAAGACCAGCTCTTCCATCGAGA | Internal control for real-time quantitative RT-PCR |
| ACTIN2 RT-RP | ACACACAAGCGCATCATAGAAACGA |
